# Metabolism of pancreatic neuroendocrine tumors: what can omics tell us?

**DOI:** 10.3389/fendo.2023.1248575

**Published:** 2023-10-16

**Authors:** Arnaud Jannin, Anne-Frédérique Dessein, Christine Do Cao, Marie-Christine Vantyghem, Benjamin Chevalier, Isabelle Van Seuningen, Nicolas Jonckheere, Lucie Coppin

**Affiliations:** ^1^ Univ. Lille, CNRS, Inserm, CHU Lille, UMR9020-U1277 - CANTHER - Cancer - Heterogeneity Plasticity and Resistance to Therapies, Lille, France; ^2^ CHU Lille, Department of Endocrinology, Diabetology, and Metabolism, Lille, France; ^3^ CHU Lille, Department of Nuclear Medicine, Lille, France

**Keywords:** pancreatic neuroendocrine tumors, metabolism, metabolomic, transcriptomic, integrative biology, MEN1, metastasis

## Abstract

**Introduction:**

Reprogramming of cellular metabolism is now a hallmark of tumorigenesis. In recent years, research on pancreatic neuroendocrine tumors (pNETs) has focused on genetic and epigenetic modifications and related signaling pathways, but few studies have been devoted to characterizing the metabolic profile of these tumors. In this review, we thoroughly investigate the metabolic pathways in pNETs by analyzing the transcriptomic and metabolomic data available in the literature.

**Methodology:**

We retrieved and downloaded gene expression profiles from all publicly available gene set enrichments (GSE43797, GSE73338, and GSE117851) to compare the differences in expressed genes based on both the stage and MEN1 mutational status. In addition, we conducted a systematic review of metabolomic data in NETs.

**Results:**

By combining transcriptomic and metabolomic approaches, we have identified a distinctive metabolism in pNETs compared with controls without pNETs. Our analysis showed dysregulations in the one-carbon, glutathione, and polyamine metabolisms, fatty acid biosynthesis, and branched-chain amino acid catabolism, which supply the tricarboxylic acid cycle. These targets are implicated in pNET cell proliferation and metastasis and could also have a prognostic impact. When analyzing the profiles of patients with or without metastasis, or with or without MEN1 mutation, we observed only a few differences due to the scarcity of published clinical data in the existing research. Consequently, further studies are now necessary to validate our data and investigate these potential targets as biomarkers or therapeutic solutions, with a specific focus on pNETs.

## 1 Introduction

It has been a century since the 1923 publication of Otto Warburg’s paper, which first described alterations in cancer metabolism compared to surrounding healthy tissue (1). Reprogramming of cellular metabolism has now become a hallmark of tumorigenesis (2–4) and has been implicated in tumor initiation, proliferation, metastasis, and chemoresistance (5–8). However, the main pathways involved in cancer metabolic plasticity may vary according to cancer type and thus require specific clarification. These aspects are particularly relevant in the case of pancreatic neuroendocrine tumors (pNETs), as only a few studies have explored their metabolism. pNETs are rare tumors, accounting for only 3%–5% of all cases of pancreatic neoplasms (9), and they originate from pancreatic hormone-producing cells (islet cells). Over the past decades, an increased incidence of pNETs has been observed due to improvements in imaging, endoscopy, and biomarker detection technologies (9–12). Despite these advances, overall survival (OS) rates have remained relatively unchanged (10).

pNETs display tumor heterogeneity depending on several factors: 1) WHO tumor grade, 2) functional status (hormone secretion), 3) stage, and 4) the genetic and epigenetic molecular signatures. For example, well-differentiated non-functional pNETs commonly have mutations in *MEN1*, *ATRX*, and *DAXX*, as well as activation of specific pathways such as mTOR, contrary to *TP53* and *RB1* mutations, which are found in more aggressive pancreatic neuroendocrine carcinomas (13–21). Many studies on pNETs have been dedicated to describing genetic or epigenetic abnormalities and their associated signaling pathways (13, 21–25). However, to the best of our knowledge, no studies have specifically investigated the metabolism of pNETs.

In addition to these now increasingly considered aspects, it is important to study the metabolism of each tumor type and identify common characteristics to establish metabolomic signatures (or biomarkers) that can predict the tumor type, its aggressiveness, and the patient’s prognostic outcome (26). A better understanding of tumor metabolism would also provide essential information for developing new therapeutic strategies, which are still lacking in this field. Several large-scale studies have used transcriptomics to compare metabolic pathways between cancer and adjacent normal tissue, as well as between different cancer types showing dysregulated genes involved in the tricarboxylic acid (TCA) cycle, oxidative phosphorylation (OXPHOS), and amino acid, fatty acid (FA), and vitamin metabolism (27, 28). The objective of this review is to describe the metabolic pathway features of pNETs by analyzing available transcriptomic and metabolomic data from the perspective of metabolic genes.

## 2 Patients and methods

### 2.1 Transcriptomic data

**Figure 1:**
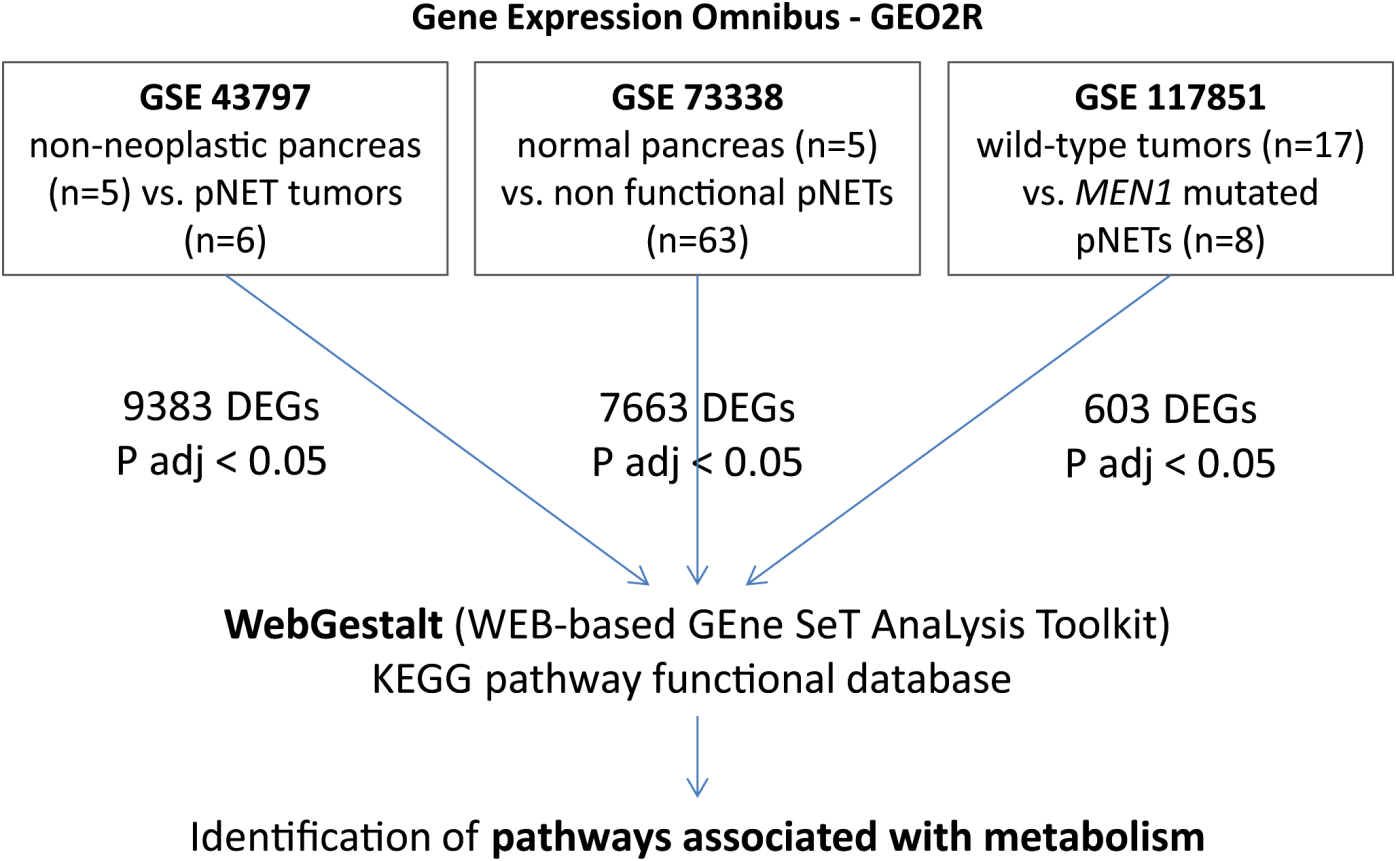
GSEA and WebGestalt analysis methodology. In GSE43797, mRNA expression in pNETs (n = 6) and non-neoplastic pancreatic tissues (n = 5). In GSE73338, we selected 63 non-functional pNETs and 5 normal pancreas samples. In GSE117851, among 47 pNET tumor specimens, 8 presented with MEN1 mutation and 17 were wild-type. Only DEGs with an adjusted p-value < 0.05 were included. GSEA, gene set enrichment analysis; pNETs, pancreatic neuroendocrine tumors; DEGs, differentially expressed genes; ADJ, adjusted; MEN1, multiple endocrine neoplasia type 1.

In the present study, we retrieved and downloaded gene expression profiles and clinical data (TMN stage and molecular profile) from all gene expression data series (GSE) datasets available in the Gene Expression Omnibus (GEO) database (http://www.ncbi.nlm.nih.gov/geo/) (GSE43797, GSE73338, and GSE117851). These datasets were used to compare differentially expressed genes (DEGs) across different pNET datasets using the GEO2R tool (**Figure 1**). Only DEGs with an adjusted p-value < 0.05 were included in the analysis.

GSE43797 was used to characterize mRNA expression in well-differentiated and non-functional pNETs (n = 6, including three with lymph node extension and two with metastasis) and non-neoplastic pancreatic tissues (n = 5 showed no evidence of chronic pancreatitis or preneoplastic lesions) (29). GSE73338 consists of a large panel of non-functional pNETs (23) (n = 63) from a global cohort that is 42% male, with an average age of 53 years (range 17–78), and an average tumor size of 30 mm. The dataset also included five normal pancreas samples without chronic pancreatitis or preneoplastic lesions (22). The *MEN1* mutation status was unknown for GSE43797 and GSE73338. GSE117851, however, consists of 47 well-differentiated pNETs of WHO grade G1/G2, including *MEN1* mutations (n = 8) or wild-type mutations (n = 17) (global cohort: 59% male, average age 52 years [range 26–73], average tumor size 36 mm) (24). The data were extracted and annotated. Functional enrichment analysis was conducted using WebGestalt (WEB-based GEne SeT AnaLysis Toolkit) (http://www.webgestalt.org/) (30). Gene set enrichment analysis was carried out with the Kyoto Encyclopedia of Genes and Genomes (KEGG) functional database, and log-fold changes were used to rank DEGs. Each target identified by KEGG and WebGestalt was subsequently studied in the literature to define its functional role.

### 2.2 Metabolomic data

Metabolites are the final products of cellular regulatory processes and are the closest -omic layer to the tumor phenotype. Their levels could thus represent the ultimate response of biological systems to genetic and environmental changes. As a result, there has been a growing interest in using metabolomics to identify biomarkers of tumor phenotypes (31, 32). As part of this study, we conducted a systematic review of the literature on metabolomic data in NET to identify dysregulated metabolites.

## 3 Results

### 3.1 Data extraction

**Figure 2:**
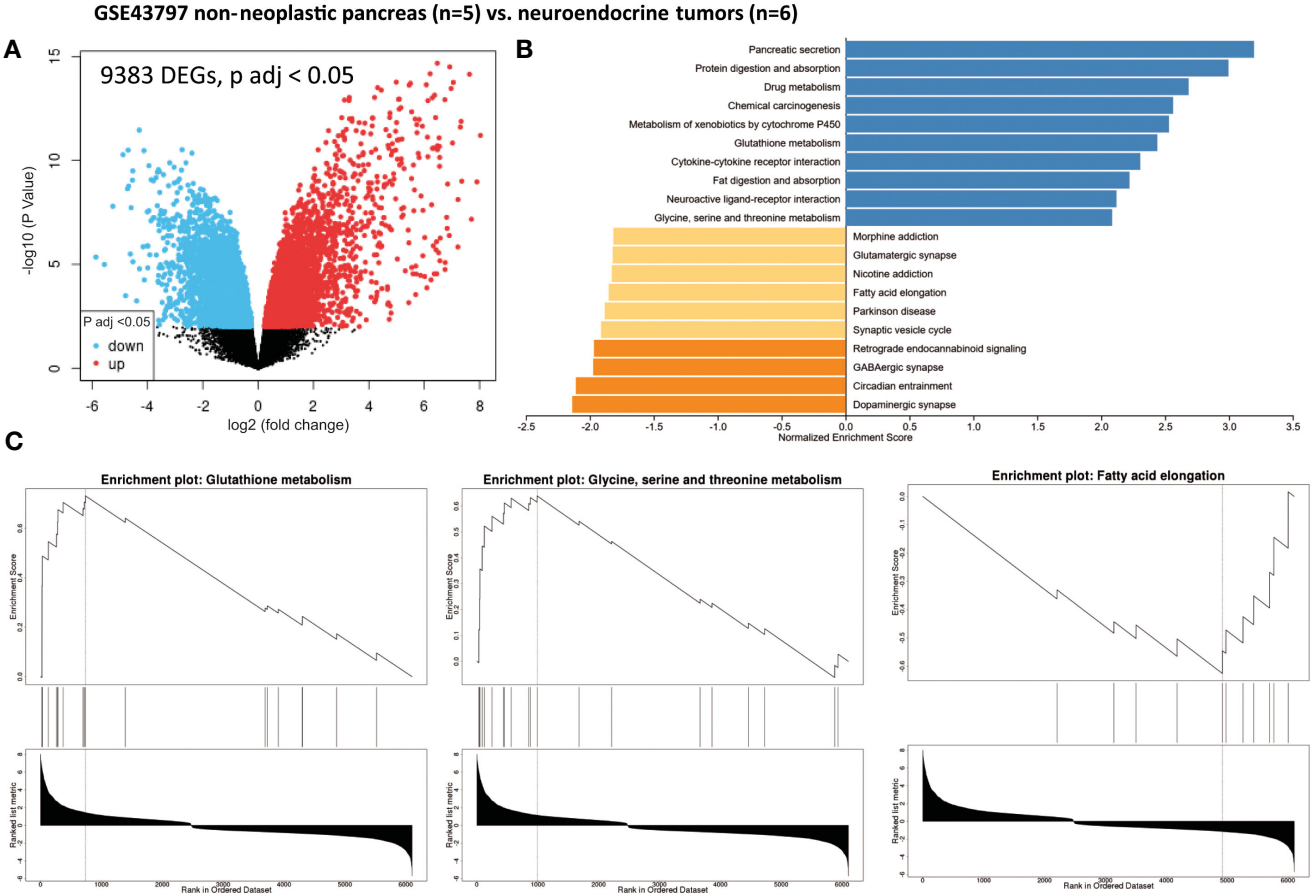
Gene set enrichment analysis (GSEA) from patients with non-neoplastic pancreas *vs.* well-differentiated and non-functional primitive pancreatic neuroendocrine tumors (GSE43797). **(A)** Volcano plot of DEGs (log2 fold change). Adjusted p-value < 0.05. **(B)** GSEA of DEGs using the WebGestalt tool. **(C)** Major enrichment GO plots corresponding to metabolism-associated KEGG term. GSEA, gene set enrichment analysis; DEGs, differentially expressed genes; GO, Gene Ontology; KEGG, Kyoto Encyclopedia of Genes and Genomes.

We identified 9,383 DEGs (adjusted p-value < 0.05) in pNETs compared with non-neoplastic pancreas samples using data from GSE43797 (**Figure 2A** and **Table 1**). We used the gene set enrichment analysis (GSEA) computational method to identify sets of enriched genes (**Figure 2B**). Notably, we observed the enrichment of Gene Ontology (GO) terms corresponding to specific functions (**Figure 2C** and **Table 1**):

- one-carbon metabolism (glycine, serine, and threonine metabolism) (*CBS*, *GNMT*, *PHGDH*, *PSAT1*, *GCAT*, *CTH*, *AMT*, *SARDH*, *SHMT1*, and *AOC3*),
- glutathione metabolism and redox balance (*GSTA2*, *GSTA1*, *ANPEP*, *GSTA5*, *GGT6*, *OPLAH*, *MGST1*, *CHAC1*, *SRM*, *GST3*, *IDH2*, and *GSTP1*),
- creatine metabolism (*GATM* and *GAMT*),
- polyamine synthesis (*SRM*),
- fatty acid metabolism and signaling (*CPT IC*, *ELOVL 4-6*, *HACD1*, *ACAA2*, *HACD3*, *ABHD17B*, and *ACLY*),
- branched-chain amino acid metabolism (valine, leucine, and isoleucine degradation) (*BCKDHA*), and
- signaling pathways (*ATF4*, *CHAC1*, and *mTOR*).

**Table 1:** Differentially expressed genes in pancreatic neuroendocrine tumors compared with non-neoplastic pancreas tissue.

Dataset	pNETs compared with non-neoplastic pancreas from GSE43797		pNETs compared with non-neoplastic pancreas from GSE73338		pNET patients with or without *MEN1* mutations from GSE117851	
Type of metabolism	Downregulated in pNET	Upregulated in pNET	Downregulated in pNET	Upregulated in pNET	Upregulated in *MEN1* pNET	Downregulated in MEN1 pNET
One-carbon metabolism	***CBS***, ***CTH***, *GNMT*, ***PHGDH***, ***PSAT1***, ***GCAT***, ***AMT***, *SARDH*, *SHMT1*	*MTRR*	***CBS***, ***CTH***, ***PHGDH***, ***GCAT***, ***PSAT1***, ***AMT***	*GSS*	*C1GALT1C1*, *HGD*, *GLS*, *GLUD2*	
Glutathione metabolism and redox balance	***GSTA2***, *GSTA1*, *ANPEP*, *GSTA5*, *GGT6*, *OPLAH*, *MGST1*, *GST3*, *IDH2*, *GSTT2B*	*GSTCD*	***GSTA2***, ***MGST1*** *GSTO4*, *GSTM4*	*GSTO1*, *GSTT2*, *GPX4*	*GPX3-4*, *GLS*	*GSTA1*
Creatine metabolism	***GATM***, ***GAMT***		***GATM***, ***GAMT***	*CKB*		
Polyamine synthesis		*SRM*				
Fatty acid metabolism and signaling		*CPT IC*, ***ELOVL 5-6***, *HACD1*, *ACAA2*, *ABHD17B*, *ACLY*	*ACADS*, *AACS*	*ELOVL5-6*, *FADS2*	*HACD1*, *ACSM3*, *DHRS3*	
Signaling pathways	*ATF4*, *CHAC1*, *mTOR*					
Branched-chain amino acid metabolism	***BCKDHB***		*ACADSB*, *BCAT1*, *ACAD8-9*, *AACS*	***BCKDHA***, *MCCC2*		
TCA and OXPHOS	*IDH2*			*OGDHL*, *IDH3*, *MDH2*	*ETFDH*	*NDUFA1,3,7*, *NDUFB11*, *NDUFS7*, *ATP6AP1*, *COX7C*, *UQCR11*, *UQCRQ*
Drug metabolism			*AOX1*, *UGT2A3*, *MGST1*, *CYP2E1*, *FMO5*, *FMO4*, *MAOB*, *CYP1A2*, *MAOA*, *ADH4*, *CYP2B6*, *ADH1B*, *FMO1*		*CYP3A5*, *CYP4F3*, *MAOA*	
cAMP signaling pathway				*GNAI1*, *ADCY2*, *GRIA2*, *GRIN2C*, *RAPGEF4*, *GNAI2*, *MAPK10*, *SSTR2*, *ATP2B1*, *ATP2A2*, *ATP1B2*, *CALM1*, *MYL9*, *ABCC4*, *EDNRA*, *TNNI3*, *RAP1A*, *ATP1A2*, *PIK3R1*, *CAMK2G*, *CREB5*		

Data from GSE43797, GSE73338, and GSE117851. Targets in black bold are expressed in GSE43797 and GSE73338.

GSE, gene set enrichment; FA, fatty acid; OXPHOS, mitochondrial oxidative phosphorylation; PPP, pentose phosphate pathway; TCA, tricarboxylic acid cycle.

Additionally, we compared pNETs from either men (n = 4) or women (n = 2) with non-neoplastic pancreatic tissue (gender not documented in GEO2R) from the GSE43797 dataset. In men, 5,801 were found to be dysregulated, while in women, 3,845 genes were dysregulated. Both lists had 3,041 DEGs in common.

**Figure 3:**
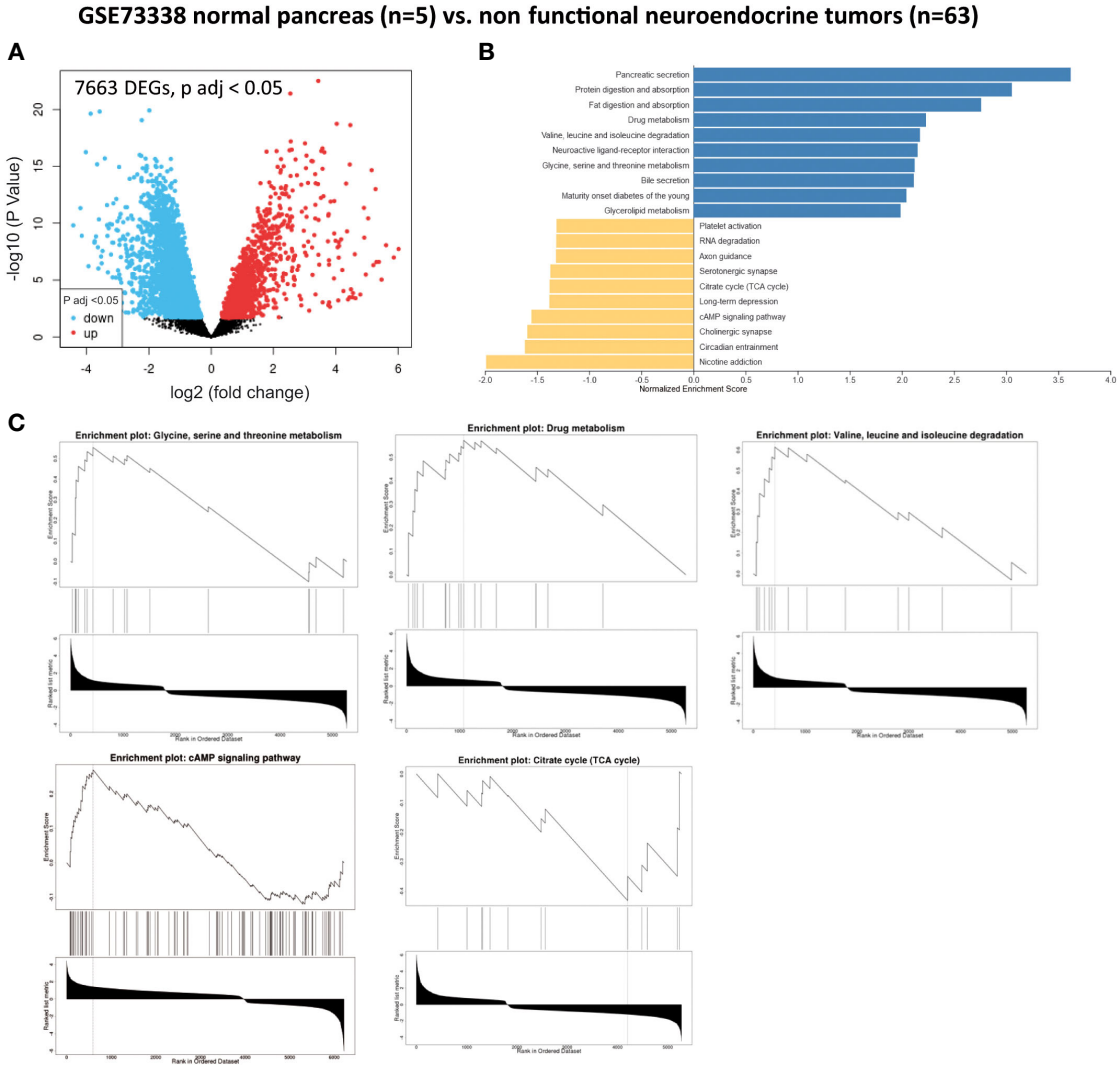
Gene set enrichment analysis (GSEA) from patients without pancreatic lesions compared with patients with well-differentiated and non-functional primitive pancreatic neuroendocrine tumors (GSE73338). **(A)** Volcano plot of DEGs (log2 fold change). Adjusted p-value < 0.05. **(B)** GSEA of DEGs using the WebGestalt tool. **(C)** Major enrichment GO plots corresponding to metabolism-associated KEGG term. GSEA, gene set enrichment analysis; DEGs, differentially expressed genes; GO, Gene Ontology; KEGG, Kyoto Encyclopedia of Genes and Genomes.

We also analyzed 63 non-functional pNET and five normal pancreas samples extracted from GSE73338. In this dataset, we identified 7,663 genes that were significantly dysregulated (adjusted p-value < 0.05) (**Figure 3A** and **Table 1**). Utilizing GSEA (**Figure 3B** and **Table 1**), we observed an enrichment of metabolism-associated GO terms, including the following (**Figure 3C** and **Table 1**):

- one-carbon metabolism (glycine, serine, and threonine metabolism) (*GATM*, *CTH*, *PHGDH*, *GCAT*, *CBS*, *PSAT1*, *GAMT*, and *AMT*),
- glutathione metabolism and redox balance (*GSTA2*, *GSTO1*, *GSTO4*, *GSTM4*, and *GPX4*),
- creatine metabolism (*GATM*, *GAMT*, and *CKB*),
- fatty acid metabolism (*ACADS*, *AACS*, and *FADS2*),
- branched-chain amino acid metabolism (valine, leucine, and isoleucine degradation) (*BCKDHA*, *ACADSB*, *BCAT1*, *AOX1*, *ACAD8*, and *MCCC2*),
- drug metabolism (*AOX1*, *UGT2A3*, *CYP2E1*, *FMO5*, *FMO4*, *MAOB*, *CYP1A2*, *MAOA*, *ADH4*, *GSTM4*, *CYP2B6*, *ADH1B*, and *FMO1*),
- cAMP signaling pathway (*GNAI1*, *ADCY2*, *GRIA2*, *GRIN2C*, *RAPGEF4*, *GNAI2*, *MAPK10*, *SSTR2*, *ATP2B1*, *ATP2A2*, *ATP1B2*, *CALM1*, *MYL9*, *ABCC4*, *EDNRA*, *TNNI3*, *RAP1A*, *ATP1A2*, *PIK3R1*, *CAMK2G*, and *CREB5*), and
- TCA cycle (*OGDHL*, *IDH3*, and *MDH2*).

In addition, we compared DEGs in pNETs versus normal pancreas samples from GSE43797 and GSE73338 and obtained a common list of 2,193 altered DEGs from both datasets.

### 3.2 Transcriptomic signature comparison of pNET patients with or without metastasis

**Figure 4:**
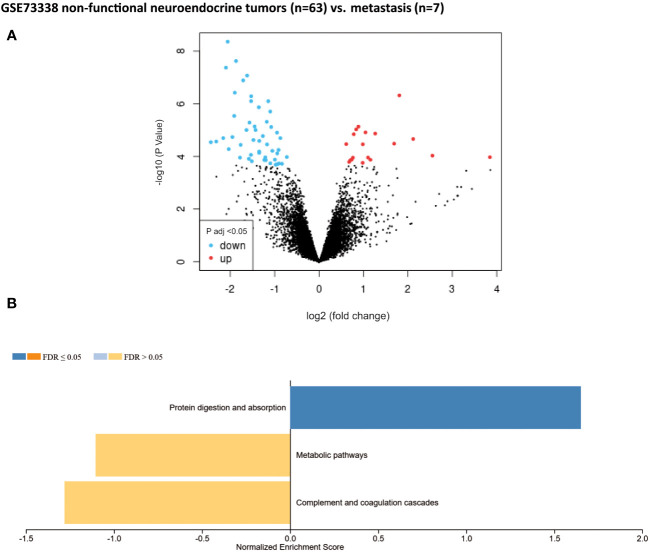
Gene set enrichment analysis (GSEA) from patients without metastasis. Well-differentiated, non-functional pNETs compared with metastatic non-functional pNETs (GSE73338). **(A)** Volcano plot of DEGs (log2 fold change). Adjusted p-value < 0.05. **(B)** GSEA of DEGs using the WebGestalt tool. DEGs, differentially expressed genes.

Non-functional pNET transcriptomic samples (n = 63) were compared with liver metastasis samples from non-functional pNETs (n = 7) using GSE73338. No differences were found in the previously mentioned major metabolic families (one-carbon metabolism, fatty acid synthesis, creatine biosynthesis, TCA, etc.). Only 69 DEGs were statistically significant (adjusted p-value < 0.05). Among them, a significant variation in targets impacted in metabolism was observed (**Figure 4**): *GYS2*, *NAT8L*, *TDO2*, *RDH16*, *DAO*, and *DPYS*.

### 3.3 Metabolic signature comparison of pNET patients with or without *MEN1* mutations

**Figure 5:**
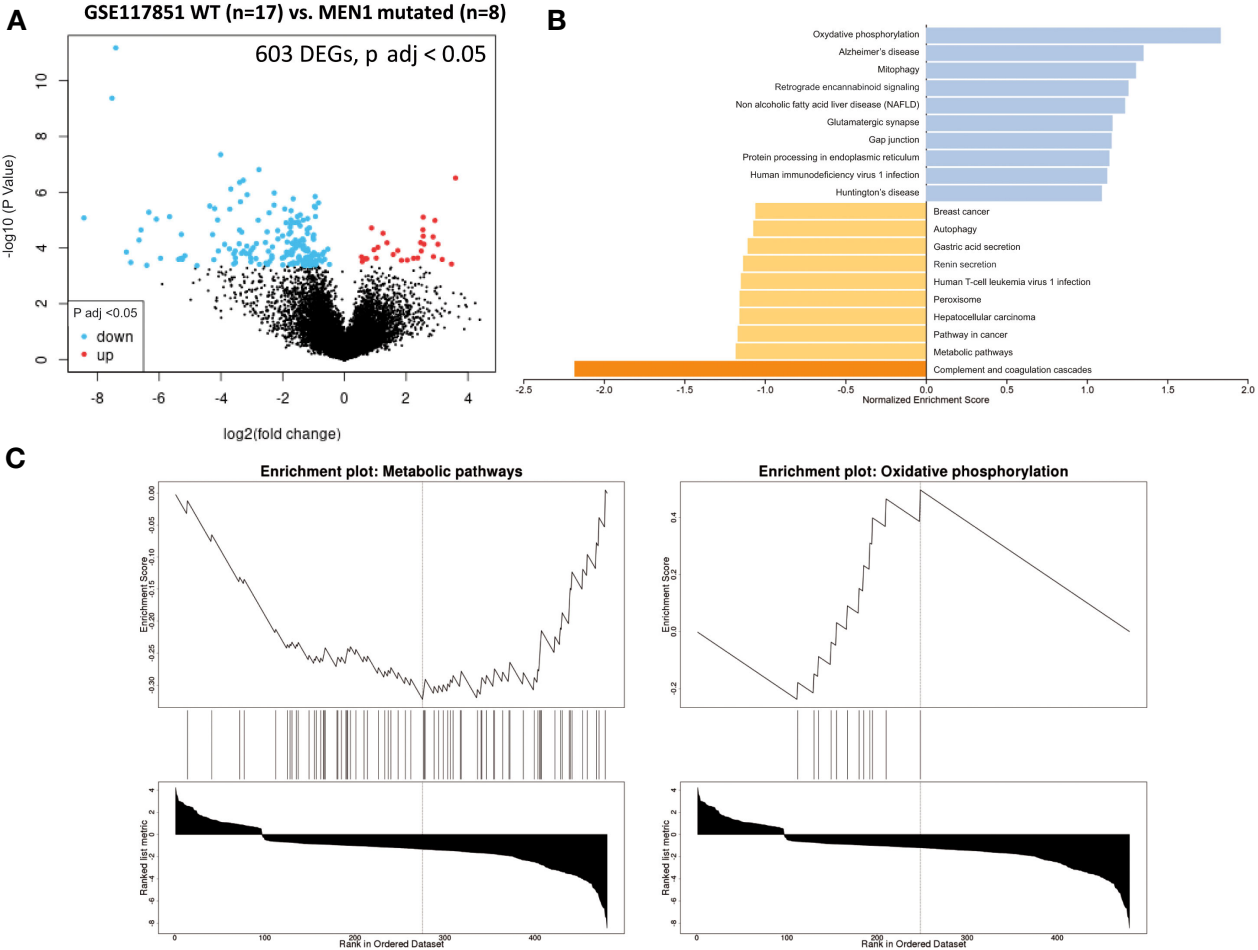
GSEA comparing pNET wild-type patients with NEM1-mutated pNET patients. Gene set enrichment analysis (GSEA) from patients with wild-type, well-differentiated, and non-functional primitive pNETs compared with NEM-mutated patients (GSE117851). **(A)** Volcano plot of DEGs (log2 fold change). Adjusted p-value < 0.05. **(B)** GSEA of DEGs using the WebGestalt tool. **(C)** Major enrichment GO plots corresponding to metabolism-associated KEGG term. GSEA, gene set enrichment analysis; DEGs, differentially expressed genes; GO, Gene Ontology; KEGG, Kyoto Encyclopedia of Genes and Genomes.

A total of 603 DEGs were extracted from GSE117851 by comparing non-functional pNETs based on whether or not they had *MEN1* mutations (**Figure 5A**). GSEA was subsequently conducted (**Figure 5B**) and showed an alteration of metabolism-associated KEGG GO terms (**Figure 5C**). In pNETs with *MEN1* mutations, an increased normalized enrichment score was observed (**Table 1**):

- one-carbon metabolism (glycine, serine, and threonine metabolism) (*C1GALT1C1*, *HGD*, *GLS*, and *GLUD2*),
- glutathione metabolism and redox balance (*GPX3-4* and *GLS*),
- fatty acid metabolism and signaling (*HACD1*, *ACSMB*, and *DHRS*), and
- other metabolic pathways (in the urea cycle (*ASS1*), tyrosine and phenylalanine (*HGD*), serine and threonine (*C1GALT1C1*), oxalate (*HAO1*), taurine (*CDO1*), choline (*PEMT*) ketogenesis (*HMGCS2*), glycerol metabolism (*GK*), amine metabolism (*MAOA*), biotin (*BTD*) and nucleotide biosynthesis (*PRPS1*), and glucose metabolism (*PCK2*)) (**Figure 5C**).

A decreased normalized enrichment score was noted in oxidative phosphorylation for *NDUFA1*, *NDUFA3*, *NDUFA7*, *NDUFB11*, *NDUFS7*, *ATP6AP1*, *COX7C*, *UQCR11*, and *UQCRQ*.

### 3.4 Metabolomic approaches in NET?

The metabolome of gastroenteropancreatic neuroendocrine tumors (GEP-NETs) has been explored by very few researchers (33–35). In 2013, Kinross et al. (**Table 2**) were the first to apply a ^1^H nuclear magnetic resonance spectroscopic profiling (^1^H NMR) technique to analyze urine from a prospective cohort of 28 patients with GEP-NETs (including 8 small intestine NETs (SI-NETs) and 10 pNETs) (33). They observed a decrease in the concentration of creatine, citrate, and hippurate in the urine of patients with GEP-NETs compared with controls. Another study by Imperiale et al. (2019) (**Table 2**) focused on SI-NETs, related hepatic metastases, and normal SI pathological tissues, using ^1^H-magic angle spinning (HRMAS) NMR spectroscopy (34). They identified and quantified 27 metabolites and observed that SI-NETs were characterized by higher concentrations of succinate, glutathione, taurine, myoinositol, and glycero-phosphocholine. SI-NET samples with aggressive profiles had lower concentrations of glucose, serine, and glycine and increased levels of choline-containing compounds, taurine, lactate, and alanine. Liver metastases were differentiated from normal hepatic parenchyma based on a higher abundance of acetate, succinate, choline, phosphocholine, taurine, lactate, and aspartate. In comparison, higher levels of alanine, ethanolamine, glycerol-phosphocholine, and glucose were found in hepatic metastases when compared with primary SI-NETs.

**Table 2:** Differentially expressed metabolites in neuroendocrine tumors compared with controls.

	NET patients compared with controls		NET patients with metastasis compared with primitive NET	
Study Sample type Pathological subtypes	Increased	Decreased	Increased	Decreased
Kinross et al., 2013 (33) Urine GEP-NET		**Creatine**, citrate, hippurate		Hippurate
Imperiale et al., 2019 (34) Tumor sample SI-NET	Succinate, glutathione, taurine, myoinositol, glycerophosphocholine	**Creatine**, alanine, ethanolamine, aspartate	Choline, glycerophosphocholine, ethanolamine, aspartate, tryptophan, isoleucine, valine, alanine, lactate, ascorbate, arginine, creatine	Serine, acetate, NAA, fumarate, tyrosine, glucose, serine glutamine
Soldevilla et al., 2021 (35) Plasma Patients with NET	Arginine, 1-methyladenosine, biliverdin, 5-hidroxyindolacetic acid, 15-hidroxyeicosatetraenoic acid, ursodeoxycholic acid, ursodeoxycholic acid 3-sulfate	Linoleoylcarnitine, oleoylcarnitine, sphingosine-1-phosphate		

Targets in bold are expressed in two distinct studies.

Finally, in a recent study, Soldevilla et al. (2021) (**Table 2**) analyzed and profiled plasma samples from 77 NET patients and 68 controls using gas chromatography–mass spectrometry (GC-MS), capillary electrophoresis–mass spectrometry (CE-MS), and liquid chromatography–mass spectrometry (LC-MS) untargeted metabolomics (35). They observed 32 enriched metabolic pathways in NET related to the TCA cycle and amino acid metabolism. Among the 32 significant pathways, the most enriched ones were as follows: arginine biosynthesis, alanine, aspartate, and glutamate metabolism; arginine and proline metabolism; glyoxylate and dicarboxylate metabolism; glutathione metabolism; aminoacyl-tRNA biosynthesis; pyruvate metabolism; and the TCA.

## 4 Discussion

In this study, we analyzed available transcriptomic and metabolomic data to investigate the role of metabolism in pNETs. Given the central role of gene expression in influencing phenotypes, transcriptome analysis provides valuable insights into phenotypic oncological variability. An important advantage over genomic or proteomic approaches is that metabolomic data include contributions from gene–environment interactions, such as drug effects, dietary influences, and microbiota activities. These factors are known to either be responsible for oncogenesis or to significantly influence the efficacy and toxicity of oncological treatments (36–39).

**Figure 6:**
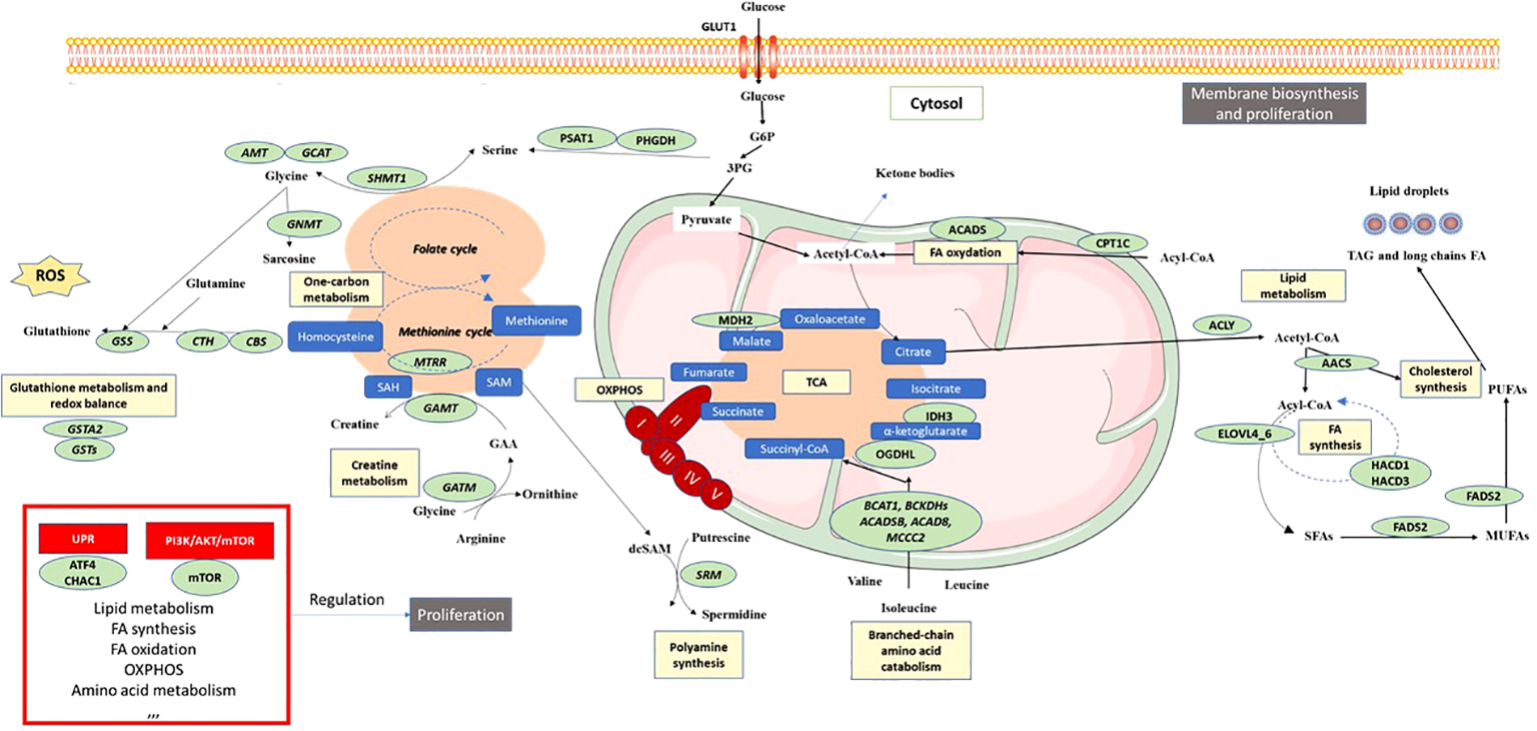
Schematic overview of pNET metabolism after integrative biology analysis. Color codes are defined as follows: green, enzymes or transporters implicated in pNET metabolism; blue, metabolites; yellow, metabolic pathway; red boxes, signaling pathways; black boxes, functional cellular mechanisms. 3PG, 3-phosphoglycerate; AOX1, aldehyde oxidase 1; AMT, aminomethyltransferase; ACLY, ATP citrate lyase; AACS, acetoacetyl-CoA-synthetase; ACADS, acyl-CoA dehydrogenase short chain; ACADSB, acyl-CoA dehydrogenase short/branched chain; ACAD8, acyl-CoA dehydrogenase family member 8; ATF4, activating transcription factor 4; BCAT1, branched chain amino acid transaminase 1; BCKDHs, branched chain keto acid dehydrogenase; CBS, cystathionine beta-synthase; CHAC1, ChaC glutathione specific gamma-glutamylcyclotransferase 1; CTH, cystathionine gamma-lyase; CPT1C, carnitine palmitoyltransferase 1C; ELOVLs, elongation of very long-chain fatty acid proteins; FADS2, fatty acid desaturase 2; GAMT, guanidinoacetate *N*-methyltransferase; GATM, glycine amidinotransferase; GLUT1, glucose transporter 1; GSS, glutathione synthetase; GSTA1,2, 5, glutathione *S*-transferase alpha 1,2,5; HACD1, 3-hydroxyacyl-CoA dehydratase 1; IDH3, isocitrate dehydrogenase 3; MCCC2, methylcrotonyl-CoA carboxylase subunit 2; MDH2, malate dehydrogenase 2; MTRR, 5-methyltetrahydrofolate-homocysteine methyltransferase reductase; mTOR, mechanistic target of rapamycin; MUFAs, monounsaturated fatty acids; NADPH, nicotinamide adenine dinucleotide phosphate; OGDHL, oxoglutarate dehydrogenase L; PHGDH, phosphoglycerate dehydrogenase; PUFAs, polyunsaturated fatty acids; ROS, reactive oxygen species; SAM, *S*-adenosyl methione; dcSAM, decarboxylated SAM; SAH, *S*-adenosylhomocysteine; SFA, saturated fatty acid; SHMT1, serine hydroxymethyltransferase 1.

The primary objective of this integrative analysis was to identify the metabolic pathways expressed differently between patients with pNET and those with healthy tissue. As expected, from a transcriptomic perspective, pNETs exhibited a loss of physiological functions, particularly related to pancreatic secretion (**Figures 2B** and **3B**). In addition, a neuronal signature was observed in pNETs, including axon guidance, serotoninergic synapse, and cholinergic synthase, validating our analyses. Furthermore, we observed a significant variation in lipid metabolism. Altered lipid metabolism is an emerging hallmark of tumors, as proliferating cancer cells undergo reprogramming of fatty acid uptake, synthesis, and storage. In addition, cancer cells need lipids for energy production and the biosynthesis of membrane components (40–42) (**Figure 6**). In the transcriptomic analysis, a significant enrichment in fatty acid synthesis (*ELOVL 4-6*, *HACD1* and *3*, *ACAA2*, *ABHD17B*, *ACLY*, and *FADS2*) in pNET tissues was observed when compared with controls (**Figures 2**, **3**, **6**, and **Table 1**). Our findings are consistent with the observations of Soldevilla et al. (2021), who identified 155 differential metabolites using a multiplatform non-targeted metabolomic approach that provided broad coverage of the metabolome. They observed that fatty acids were the second most abundant biochemical class following amino acid derivatives (35). In addition to a family history of NET, epidemiological evidence from observational studies suggests that a higher body mass index and type 2 diabetes are important risk factors for NET oncogenesis, independent of the NET origin (43–46). However, to the best of our knowledge, no study has focused on the link between obesity, pancreatic fatty acid infiltration, and progression-free survival (PFS) or OS in pNETs. In challenging microenvironment conditions, such as those in oncogenesis, acetyl-CoA upregulates fatty acid synthesis to create favorable conditions for cell survival, proliferation, metastasis, and stress resistance (40, 47). Fatty acid metabolism should therefore be considered a valid target in pNET patients with this profile. For example, targeting FASN (with the FASN inhibitor TVB-2640) or ACLY (with the acyl inhibitor SB-204990) has shown anticancer effects in some preclinical models, and this approach is currently being tested as anticancer therapies in clinical trials (48). Interestingly, Orlistat, a FASN inhibitor, has been shown to inhibit the progression of pNETs by inducing ferroptosis (49).

Another significant aspect of metabolic cancer cell reprogramming is one-carbon metabolism, which is useful for methylation reactions, the generation of reducing cofactors, and nucleotide and creatine biosynthesis (50–52). In the two GSE datasets comparing normal and pNET samples, enrichment of targets involved in one-carbon metabolism was observed, particularly those involved in serine synthesis (*PHGDH* and *PSAT1*), glycine synthesis (*GCAT AMT*), and glutathione synthesis (*CBS* and *CTH*). Interestingly, Soldevilla et al. (2021) showed increased plasma concentrations of leucine, glycine, and serine in patients with NETs compared with controls (**Table 2**) (35). Excessive activation of serine/glycine biosynthesis drives tumorigenesis and provides a single-carbon unit for one-carbon metabolism and the necessary creatine biosynthesis. Interestingly, both Kinross et al. (2013) and Imperiale et al. (2019) have independently shown, using different methodological analyses, that the level of creatine was lower in NET samples (urine and tumor samples) from patients with NETs (**Table 2**) (33, 34). Considering the importance of creatine metabolism in cancer cell survival, metastasis (through smad2/3), and immune evasion, it could be a potential target in pNET (53, 54). The activation of one-carbon metabolism also involves the methionine cycle and thus polyamine biosynthesis, wherein one of the key enzymes (*SRM*) was found to be upregulated in GSE43797 (**Table 1** and **Figure 6**). This finding is consistent with the plasma metabolomic analysis carried out by Soldevilla et al. (2021), where an increase in acetylspermidine polyamine was observed, illustrating the relevance of polyamine metabolism in NETs. Furthermore, glutathione metabolism, which is also influenced by one-carbon metabolism, plays a vital role in providing a stronger antioxidant capacity to survive in a more oxidative environment due to the sharp rise in ROS generation in cancer cells (55). Our analysis of pNET patients compared with controls also showed dysregulation of glutathione metabolism targets (*GSTA2*, various *GSTs*, and *GPX4*) (**Figure 6**). These enzymes are useful for protecting cancer cells against H_2_O_2_-induced cell death and ferroptosis, as they compensate for the elevated ROS stress and confer resistance to a number of chemotherapeutic agents (49, 56–59). Interestingly, glutathione was found to be characteristic and more abundant in SI-NETs compared with normal small intestine tissue in the Imperiale study and in GEP-NET plasma samples in the Soldevilla study (34, 35). This suggests that higher glutathione metabolism may be present in GEP-NETs and therefore appears to be a pathway of interest.

Finally, in our analysis, we observed TCA cycle alterations in pNET and GEP-NET when compared with control patients without NETs whether in transcriptomic analyses (*OGDHL*, *IDH3*, and *MDH1*) or metabolomic approaches (succinate or isocitrate/citrate alterations) (**Figures 2**, **3**, **6**, and **Table 1**). Metabolism fueling the TCA cycle, such as fatty acid oxidation and branched-chain amino acid (alanine, leucine, and isoleucine) metabolism, were also found to be altered. In terms of fatty acid oxidation, we observed an increase in *CPT1* and *ACADS* expression in the two GSE datasets comparing normal pancreas and pNETs, contributing to an increase in acetyl-CoA, which provides an energy supply for the TCA cycle. After transcriptomic analysis, branched-chain amino acid targets were also increased in pNET samples (*BCKDHB*). These branched-chain amino acids are metabolized in the mitochondria into ketoacids, generating succinyl CoA and acetyl-CoA for oxidation by the TCA cycle (**Figure 6**) (60). Describing the alterations of the TCA cycle in NETs more precisely is important to better understand the oncogenesis of these tumors and to consider new therapeutic approaches that directly target these metabolites (61).

The second objective was to identify the metabolic pathways expressed differently between primary pNETs and metastasis. Unfortunately, only one GSE dataset, which included a limited number of metastatic patients, was available for this analysis, therefore yielding few results. Despite this limitation, we observed little difference between the primitive pNET tissues and metastatic pNET tissues, which may suggest common metabolic characteristics. We only observed enrichment in targets implicated in amino acid metabolism, notably with regard to tryptophan (*TDO2*, *DAO*, and *DPYS*). This finding was also confirmed in the study by Imperiale et al. (2019), where tryptophan was found to be higher in liver metastasis compared with primitive SI-NETs. It is well-known that tumors use tryptophan and its metabolites to promote their growth and metastasis and evade host defenses. Therefore, studying this pathway in pNETs could be particularly interesting (62, 63). Additionally, fatty acid metabolism (*RDH16*) was also found to be upregulated in our study. Imperiale et al. (2019) also observed higher levels of acetate, which can be considered an index of fatty acid synthesis following beta-oxidation of acetyl-CoA, in aggressive SI-NETs, thus confirming the significance of fatty acid metabolism in NETs as well as in different cancers (34, 64, 65). Moreover, an enrichment in other metabolomic targets was identified (*GYS2* and *NAT8L*), which are potential targets as in other cancers (66–69).

Our last objective was to identify the metabolic pathways that are differently expressed in pNET patients based on the presence or absence of germinal *MEN1* mutation. Interestingly, we observed a differential expression in mRNA implicated in OXPHOS (*NDUFA1*, *NDUFA3*, *NDUFA7*, *NDUFB11*, *NDUFS7*, *ATP6AP1*, *COX7C*, *UQCR11*, *UQCRQ*, and *NAMPT*). We found an association between *MEN1* status and genes involved in OXPHOS. This association was further supported by a recent article demonstrating that *MEN1* regulates the expression of genes involved in OXPHOS and glycolysis to coordinate cellular response for energy production (70). To the best of our knowledge, no metabolomic study has addressed this question, but it is an important research point that should be developed further to better understand the differences between pNETs with and without *MEN1* mutations. Recently, Fahrmann et al. reported a plasma acetylated polyamine metabolite signature (*N*-acetylputrescine, acetylspermidine, and diacetylspermidine) associated with MEN1-duodenopancreatic NET disease progression. However, no overexpression of polyamine pathway mRNAs was observed in metastatic MEN1 patients (71, 72).

Using duodenopancreatic NET transcriptomic analysis to focus on prognosis, as was done in the Diedisheim study (25), we observed that the targets associated with TCA were associated with poor (*OGDHL*) or intermediate (*IDH* and *MDH1*) outcomes. Targets implicated in fatty acid metabolism were associated with both poor outcomes (*FASN*) and better outcomes (*ELOVL4*, *ACLY*, and *AACS*). Targets implicated in amino acid metabolism were associated with poor outcomes (*SHMT1*), intermediate outcomes (*GATM*, *CBS*, *GNMT*, *PHGDH*, *AMT*, *GCAT*, *AOC3*, *BCAT1*, and *ACADS*), or better outcomes (*AOX1* and *AACS*). Targets implicated in glutathione metabolism were associated with poor outcomes (*GSTA1* and *MGST1*) and intermediate outcomes (*GSTA2*, *ANPEP*, *OPLAH*, *SRM*, *IDH2*, *GSTP1*, *GSTO1*, and *MAOB*). Similarly, a recent proteo-transcriptomic classification of pNETs showed that several different metabolic targets were overexpressed in the pNET proliferative subgroup: *PHGDH*, *SDMT1*, *MGST1*, and *FASN* (73).

Our original analysis suggests that integrating transcriptomics with metabolomics can provide insights into how metabolites are regulated and can elucidate targetable functional mechanisms. However, this study has several limitations. First, RNA sequencing approaches allow comparison of the quantitative expression of the genes of interest and can therefore provide an idea of intratumoral metabolism. However, they do not precisely describe its functioning, as they completely ignore the influence of epigenetics and other mechanisms that can impact the functioning of the studied enzymes. Additionally, these approaches do not take into account the metabolism of non-cancer stromal and immune cells.

Second, metabolites represent the end product of gene expression. Metabolomic analysis is therefore the most precise methodology for defining the metabolic functioning of pNETs. However, the only publications using metabolomic analysis were from studies conducted in all types of NETs and not specifically in pNETs, despite the well-known importance of the primitive organ in defining the metabolic nature of the tumor, which also depends on the tumor grade; these details were not described in these GSE datasets (74). In addition, these metabolomic studies used various sample types, including tumor, urine, and plasma, which provided different sets of information. To comprehensively understand the molecular heterogeneity in pNETs (75, 76), further studies combining single-cell RNA sequencing and metabolomic approaches in tumor samples are necessary, as has been previously suggested (7, 23).

Integrative biology, combining transcriptomic and metabolomic approaches, demonstrates a distinct metabolic profile in pNETs characterized by dysregulation of one-carbon metabolism and glutathione metabolism, and fatty acid biosynthesis, which facilitate cancer cell proliferation as well as fatty acid oxidation and branched-chain amino acid catabolism, which supply the tricarboxylic acid cycle.

These targets are implicated in signaling pathways such as the unfolded protein response and mTOR pathways, which are also associated with pNET cell proliferation and metastasis. The targets highlighted in this study make it possible to differentiate patients with or without pNETs and could also serve as prognostic indicators. However, further studies are now needed to validate these findings and to explore these targets as potential biomarkers or therapeutic options specifically tailored for pNETs.

## Author contributions

AJ, A-FD, NJ, and LC wrote and proofread the manuscript and created the figures. CDC, M-CV, BC, and IVS proofread and corrected the manuscript. AJ has full access to all the data in this study and takes responsibility for the integrity of the data and the accuracy of the data analysis. All authors have read and approved the submitted version.
